# RACIAL/ETHNIC COGNITIVE HEALTH DISPARITIES: THE ROLE OF OCCUPATIONAL COMPLEXITY AND OCCUPATIONAL STATUS

**DOI:** 10.1093/geroni/igad104.0861

**Published:** 2023-12-21

**Authors:** Mara Sheftel, Noreen Goldman, Anne Pebley, Borianna Pratt, Sung Park

**Affiliations:** Penn State University, State College, Pennsylvania, United States; Princeton University, Princeton, New Jersey, United States; University of California, Los Angeles, Los Angeles, California, United States; Princeton University, Princeton, New Jersey, United States; University of Massachusetts Boston, Boston, Massachusetts, United States

## Abstract

High levels of labor market segregation mean that Black and Latino workers may not have the same exposure to jobs involving complex work as their White counterparts, an aspect of work that appears to be protective of older adult cognition. The association between cognitive disparities and variation in exposure to occupational complexity by race/ethnicity remains understudied. Using work histories constructed from the Health and Retirement Study (HRS) life history data we analyze the role of occupational complexity in the development of dementia at older ages and address a conjecture that complexity reflects occupational status. Findings highlight that: (1) occupations involving complex work with data during working ages may be protective against dementia at older ages, potentially contributing to the differentials in dementia prevalence by race/ethnicity, and (2) occupational complexity reflects occupational status. This research increases understanding of the implications of labor market segregation for cognitive health disparities by race/ethnicity.

